# Detection of chromosome structural variation by targeted next-generation sequencing and a deep learning application

**DOI:** 10.1038/s41598-019-40364-5

**Published:** 2019-03-06

**Authors:** Hosub Park, Sung-Min Chun, Jooyong Shim, Ji-Hye Oh, Eun Jeong Cho, Hee Sang Hwang, Ji-Young Lee, Deokhoon Kim, Se Jin Jang, Soo Jeong Nam, Changha Hwang, Insuk Sohn, Chang Ohk Sung

**Affiliations:** 1 Department of Pathology, University of Ulsan College of Medicine, Asan Medical Center, Seoul, Korea; 2 Center for Cancer Genome Discovery, Asan Institute for Life Science, University of Ulsan College of Medicine, Asan Medical Center, Seoul, Korea; 3 Institute of Statistical Information, Department of Statistics, Inje University, Gyeongsangnam-do, Korea; 4 Department of Medical Science, Asan Medical Institute of Convergence Science and Technology, University of Ulsan College of Medicine, Asan Medical Center, Seoul, Korea; 5 Department of Applied Statistics, Dankook University, Gyeonggido, Korea; 6 Biostatistics and Clinical Epidemiology Center, Research Institute for Future Medicine, Samsung Medical Center, Seoul, Korea

## Abstract

Molecular testing is increasingly important in cancer diagnosis. Targeted next generation sequencing (NGS) is widely accepted method but structural variation (SV) detection by targeted NGS remains challenging. In the brain tumor, identification of molecular alterations, including 1p/19q co-deletion, is essential for accurate glial tumor classification. Hence, we used targeted NGS to detect 1p/19q co-deletion using a newly developed deep learning (DL) model in 61 tumors, including 19 oligodendroglial tumors. An ensemble 1-dimentional convolution neural network was developed and used to detect the 1p/19q co-deletion. External validation was performed using 427 low-grade glial tumors from The Cancer Genome Atlas (TCGA). Manual review of the copy number plot from the targeted NGS identified the 1p/19q co-deletion in all 19 oligodendroglial tumors. Our DL model also perfectly detected the 1p/19q co-deletion (area under the curve, AUC = 1) in the testing set, and yielded reproducible results (AUC = 0.9652) in the validation set (n = 427), although the validation data were generated on a completely different platform (SNP Array 6.0 platform). In conclusion, targeted NGS using a cancer gene panel is a promising approach for classifying glial tumors, and DL can be successfully integrated for the SV detection in NGS data.

## Introduction

The paradigm of cancer diagnosis has recently changed from a histology-based approach to a strategy that combines histology and molecular features. In particular, molecular testing has become essential for diagnosis of tumors of the central nervous system^1^. For example, to diagnose oligodendroglial tumors, mutation in the *IDH* gene and co-deletion of 1p/19q must be detected. Currently, fluorescence *in situ* hybridization (FISH) is the gold standard method for detecting 1p/19q co-deletion^2–6^; however, this test can yield false positive results because FISH probes cannot cover the whole chromosome arm^7^; moreover, interpretation is time-consuming and labor-intensive.

Currently, next-generation sequencing (NGS) using a targeted cancer gene panel has become a popular diagnostic method, and has been adopted in many hospitals^8^. However, clinical NGS testing focuses mainly on detection of mutations such as single-nucleotide polymorphisms (SNPs) and small indels. Although copy number variation (CNV) can also be detected by targeted NGS, and a few software packages have been developed for this purpose^9–11^, CNV detection via NGS remains challenging, and manual reviews of CNV plots are required for accurate results. The 1p/19q co-deletion associated with oligodendroglial tumor is characterized by simultaneous whole-arm deletion of chromosome 1 and chromosome 19; therefore, arm-level evaluation is required. However, detection of such large deletion is not well supported by CNV detection programs for targeted NGS. In addition, using only tumor tissue without matched normal tissue makes it difficult to accurately detect CNV in clinical targeted NGS tests. Therefore, it is necessary to evaluate whether targeted NGS can detect 1p/19q co-deletion with sufficient accuracy for clinical use. If targeted NGS could be used to detect 1p/19q co-deletion as well as *IDH* mutation, it would have marked advantages over standard molecular tests such as FISH, immunohistochemistry, and Sanger sequencing.

Currently, many researchers are investigating the application of deep learning (DL) into various diagnostic areas. Many studies of DL have focused on diagnosis using histologic and radiologic images. Recently, however, DL has been applied to analysis of sequencing to improve the sensitivity and specificity of SNP and indel detection^12^. We speculated that CNV detection would also be a good candidate for application of DL. Hence, we developed a DL model for analyzing copy number variation in NGS data from a targeted cancer panel, with the goal of detecting 1p/19q co-deletion.

## Results

### Summary of targeted NGS testing and diagnostic performance

**Figure 1:**
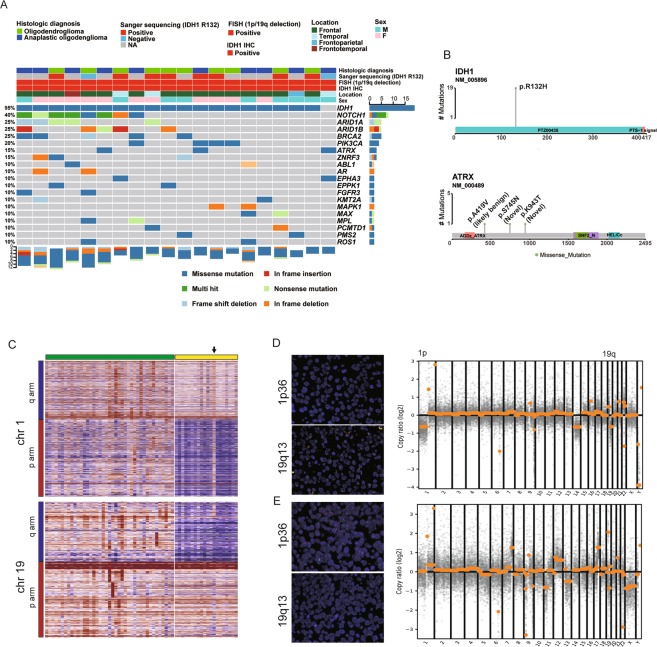
Summary of detected variants using targeted next generation sequencing (NGS) in an initial set of 20 enrolled tumors with various clinicopathological factors (**A**). Mutations with protein annotation of *IDH1* and *ATRX* (**B**). Copy number heatmap for 1p and 19q in 20 enrolled cases (red bar) and 41 controlled cases with no 1p/19q deletion (green bar) (**C**). Representative 1p/19q co-deletion in fluorescence *in situ* hybridization (FISH) and copy number variation (CNV) plot of targeted NGS (**D**), and one false-positive case **(E**).

Single gene–based molecular test results for initial 20 cases, including evaluation of *IDH1* mutation status by Sanger sequencing and immunohistochemistry and 1p/19q co-deletion status by FISH, are shown in Fig. 1A. Primary histologic diagnosis was based on histology and single gene–based molecular tests. Among the 20 cases, 2 were interpreted as negative for *IDH1* mutation by Sanger sequencing, but targeted NGS testing revealed the R132H mutation in one of them (Fig. 1B). The other case that was negative in Sanger sequencing was also negative in targeted NGS testing. Thus, Sanger sequencing yielded a false negative result in one case. The other 18 cases were concordant between Sanger sequencing and targeted NGS testing for positive *IDH* mutation. *ATRX* mutations were found in three samples, but these mutations are not known to be of biological significance (Fig. 1B).

Manual review of the copy number plot from the targeted NGS testing identified 1p/19q co-deletion in 19 of 20 cases (Fig. 1C, yellow bar, and Fig. 1D). One case harbored only partial deletion of 19q and no 1p deletion (Fig. 1C, arrow), and review of the FISH results by experts (S.J.N. and C.O.S.) suggested false positive (Fig. 1E). This case was also negative for *IDH* mutation in both Sanger sequencing and NGS testing. Therefore, this case was excluded for further analysis. None of the negative control samples harbored 1p/19q co-deletion (Fig. 1C, green bar). Overall, these findings demonstrate that targeted NGS testing can detect 1p/19q co-deletion and *IDH* gene mutation with high accuracy.

### Mutations according to histologic grade of oligodendroglial tumors

**Figure 2:**
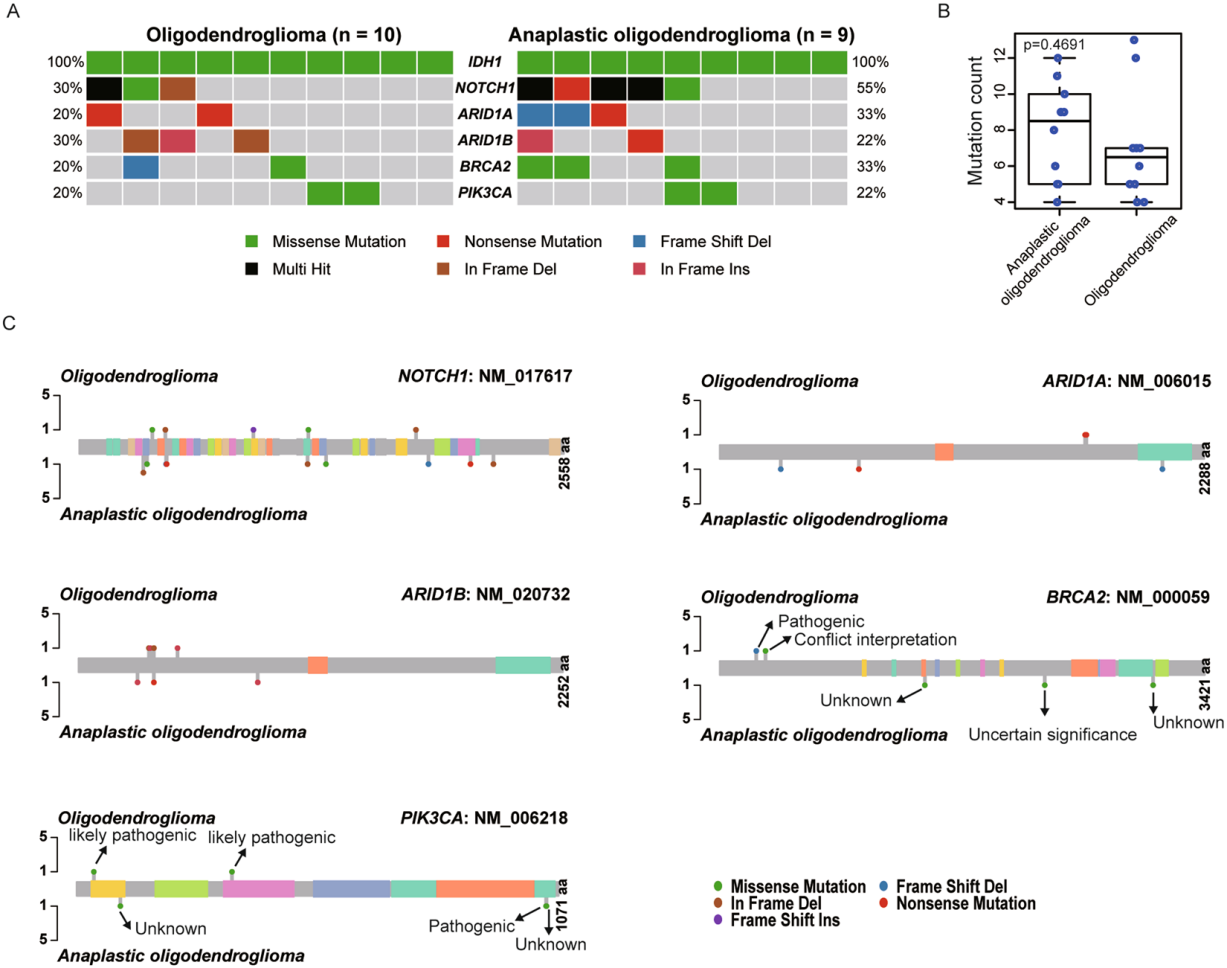
Major variants detected in 19 oligodendroglial tumors, including 10 oligodendroglioma and 9 anaplastic oligodendroglioma (**A**). Total mutation counts in oligodendroglioma and anaplastic oligodendroglioma (**B**). Mutation locus of each variant between oligodendroglioma and anaplastic oligodendroglioma (**C**).

Oligodendroglioma (WHO grade II) and anaplastic oligodendroglioma (WHO grade III) have different histologic features and clinical behaviors^1^; therefore, it may be interesting to know how mutational features in this data set correlate with histologic grade. Thus, we additionally analyzed mutation features according to histologic grade. In addition to *IDH1*, frequently mutated genes included *NOTCH1* (42.1%), *ARID1A* (26.3%), *ARID1B* (26.3%), *BRCA2* (26.3%), and *PIK3CA* (21.0%), but mutation frequencies (Fig. 2A) and mutation number (Fig. 2B) did not differ significantly between oligodendroglioma and anaplastic oligodendroglioma. Details of the mutations in these five genes are shown in Fig. 2C. Interestingly, one pathogenic *BRCA2* mutation (p.N257Kfs*17; rs80359671) was present, but the other *BRCA2* mutations were of uncertain significance or their biological significance was previously unreported.

### Development of ensemble one-dimensional convolution neural network for detection of 1p/19q co-deletion

**Figure 3:**
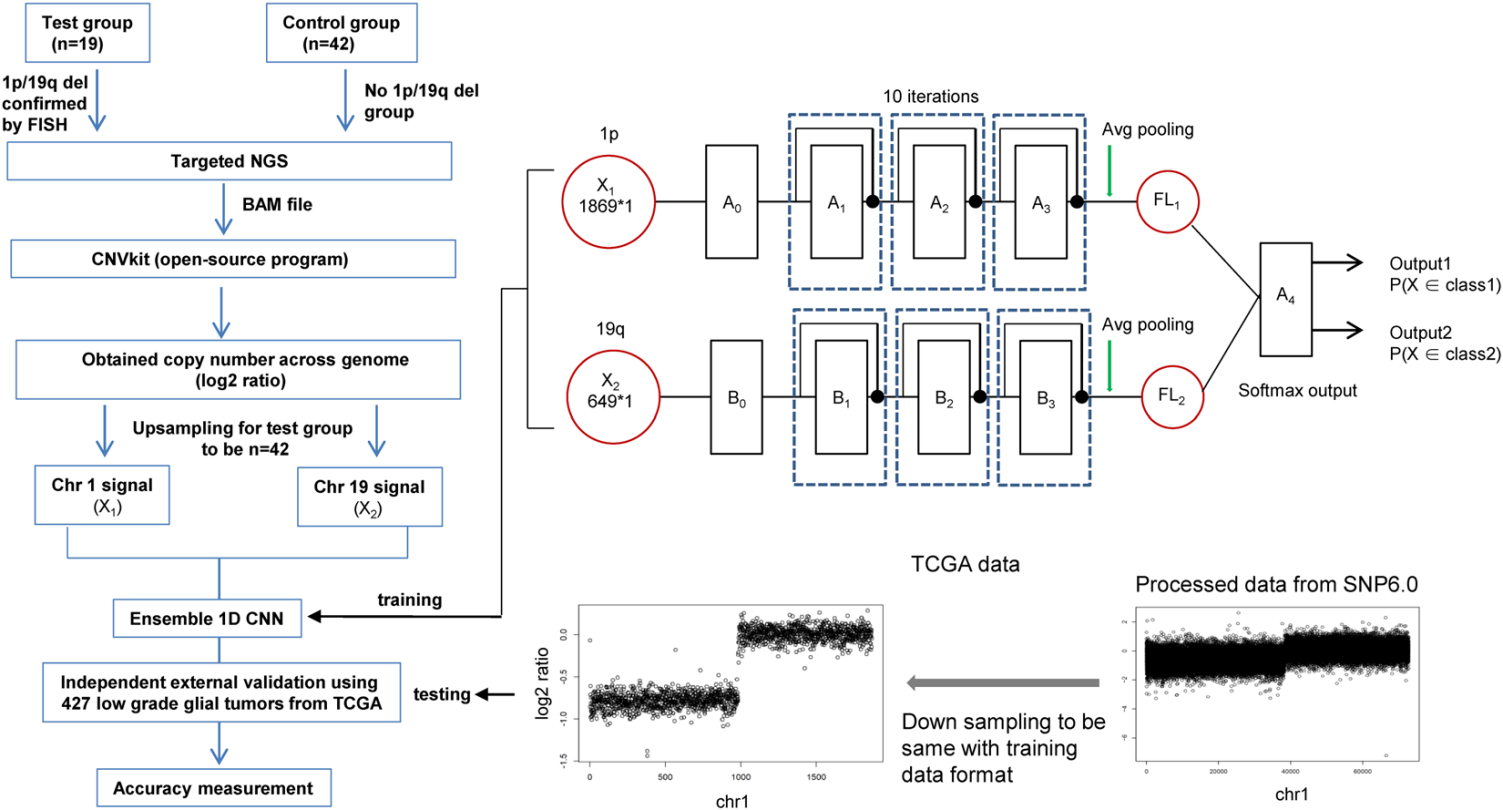
Established pipeline for detection of 1p/19q co-deletion using ensemble 1D convolutional neural network (1D CNN) from targeted next generation sequencing (NGS) data and external validation using copy number data of 427 low grade glial tumors from SNP Array 6.0 platform downloaded from TCGA. A_0_ denotes the pretraining block, whose architecture is (170*1 convolution, 32)-(2*1 max pool)-(151*1 convolution, 64)-(2*1 max pool)-(51*1 convolution, 64)-(2*1 max pool), where (170*1 convolution, 32) indicates a convolution layer composed of 32 kernels with height = 170 and width = 1, and (2*1 max pool) indicates a max pooling layer of pooling windows with height = 2 and width = 1. B_0_ denotes the pretraining block, whose architecture is (150*1 convolution, 32)-(2*1 max pool)-(51*1 convolution, 64)-(2*1 max pool)-(51*1 convolution, 64)-(2*1 max pool). A_1_ denotes the first residual block, whose architecture is (1*1 convolution, 32)-(3*1 convolution, 32)-(1*1 convolution, 128); A_2_ the second residual block, whose architecture is (1*1 convolution, 64)-(3*1 convolution, 64)-(1*1 convolution, 256); and A_3_ the third residual block, whose architecture is (1*1 convolution, 128)-(3*1 convolution, 128)-(1*1 convolution, 512). The dotted rectangle implies that the procedures inside are executed over 10 iterations. FL_1_ and FL_2_ denote the 1024*1 flattened vectors. A_4_ denotes the 2048*2 softmax output layer. Output1 and Output2 indicate outputs from the softmax output layer, whose values are P(X ∈ class1) and P(X ∈ class2), respectively, where X = X1 ∪ X2. Classes 1 and 2 indicate 1p/19q co-deletion and no 1p/19q co-deletion, respectively.

Finally, 19 cases with 1p/19q co-deletion and 42 control cases lacking such a deletion were used as a test group for model building. To address the group unbalance problem between test and control groups, the test group was up-sampled using SOMTE (Synthetic Minority Over-sampling Technique)^13^ to 42 to match the number in the control group. Log_2_ copy number ratios for chr1 and chr19 were extracted from whole chromosomes, and the signals were converted to 1869 × 1 (X_1_) and 649 × 1 (X_2_) vectors, respectively. These two data sets (chr1 andchr19) have completely different input variables. We used the ensemble method, a technique that creates multiple models and then combines them to obtain improved results, for the two data sets illustrated in Fig. 3. For the proposed ensemble model, we first applied a one-dimensional convolution neural network (1D-CNN) to the corresponding data set and obtained feature values for all cases after the uppermost pooling layer. We then concatenated these feature values and utilized them as input values of a fully connected neural network equipped with a softmax output layer for classification. External validation of this model was performed using 427 low-grade glial tumors from The Cancer Genome Atlas (TCGA) data. These data were generated using Affymetrix SNP array 6.0 platform. The log_2_ ratios were calculated from tumor and matched normal tissue, and then the log_2_ data were down-sampled to the same vector size used in the training model. The overall workflow is summarized in Fig. 3.

### Diagnostic accuracy of the DL model established using ensemble 1D-CNN

**Figure 4:**
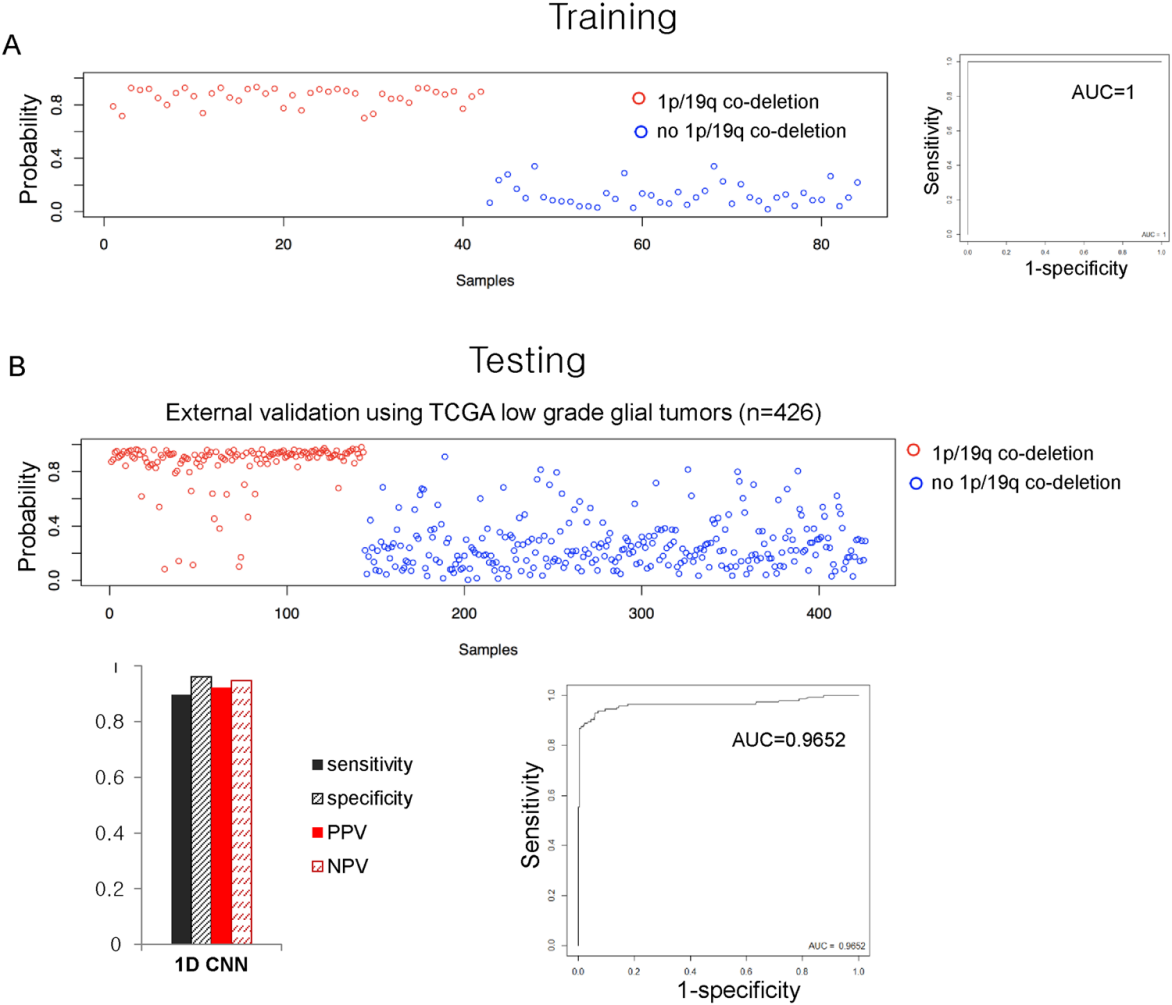
Accuracy of deep learning model for detection of 1p/19q co-deletion. The accuracy of deep learning model in the training set to detect 1p/19q co-deletion from targeted next generation sequencing data was 100% (**A**). This model was externally validated using copy number data from the SNP Array 6.0 platform downloaded from TCGA and area under the curve (AUC) value was measured as 0.9652.

The build model was trained using the AMC (Asan Medical Center, Seoul, Korea) data set generated by targeted NGS. For detection of 1p/19q co-deletion, the area under the curve (AUC) of the trained DL model was 1 (Fig. 4A). The trained model was then tested using an independent data set consisting of 426 low-grade glial tumors from TCGA, processed as described above. The optimal cut-off point was determined by maximizing the Youden index (Sensitivity + Specificity − 1) based on the predicted probability of training data set, and then the optimal cut-off was used to calculate the sensitivity and specificity of the validation set (0.8951 and 0.9611, respectively; AUC, 0.9652) (Fig. 4B).

## Discussion

In this study, we demonstrated that targeted NGS testing can accurately detect 1p/19q co-deletion. In addition, NGS testing has a comparative advantage in IDH gene detection: namely, NGS testing has greater sensitivity than Sanger sequencing and can cover the entire exome of the *IDH1* and *IDH2* genes. Recent studies also showed that targeted NGS testing is a promising diagnostic method for classifying glial tumors of the CNS^14,15^. However, the cancer gene panels used for targeted NGS are diverse, and different panels are used by various hospitals. To detect CNV by targeted NGS, a cancer panel must be designed to cover the whole chromosome, and internal controls to adjust for uneven amplification in NGS may be required. The cancer panel used in this study included hot spot mutations that occur frequently in the Korean population, but did not have an internal control to adjust CNV. Nonetheless, NGS testing using this cancer panel correctly detected 1p/19q co-deletion.

Recently, DL has been applied to diagnostics, focusing on imaging-based diagnoses such as mitotic count evaluation^16,17^ and detection of metastatic tumor in sentinel lymph nodes^18^. DL has the potential to be applied to all types of medical images, including radiologic imaging, EKG imaging, and photographs of skin lesions. However, the most important problem to overcome in this context is low accuracy in external validation. This accuracy issue can be attributed to many factors, including differences in background image color, inaccurate annotation of the target area, and heterogeneity of the target area. Therefore, images generated by computing are good candidates for application of DL. Recent studies reported major improvements in SNP/indel detection from NGS genomic data^12^. In this study, we showed that CNV from targeted NGS data can also be accurately detected using DL, even with a small training sample size. This was possible because all copy number data were generated in a regular format using a computational approach and the distinctive features of 1p/19q co-deletion in the CNV plot image. For external validation, we used CNV data from the SNP Array 6.0 platform. Even though completely different platforms were used to generate the training and validation sets, we achieved good accuracy in the validation set despite the small number of samples used for training.

Initially, we used a CNN with a whole CNV plot image to detect 1p/19q co-deletion, but this approach was associated with an unacceptably large computational workload. In addition, simultaneous detection of each 1p and 19q deletion is needed to detect the 1p/19q co-deletion, because this co-deletion is required for diagnosis of oligodendroglioma. In this study, we developed a new ensemble 1D-CNN method for detecting 1p/19q co-deletion from CNV data. The two input data sets for 1p and 19q have completely different input variables. For classification, we could combine the two data sets into a single data set and apply a classifier to these data, but to achieve better results, we used an ensemble method. Although the model was developed for the detection of the 1p/19q co-deletion, it could also be used to detect other chromosomal structural variations with similar patterns, such as arm level broad copy number amplification and/or deletion in other chromosomal loci, irrespective of tumor type. However, in this study, we did not test the detection ability of multiple forms of 1p/19q co-deletion, such as partial deletions, because our test group was small in number and did not include several multiple forms of partial deletions.

In this analysis, the relative order of bins on a chromosome was used to summarize the chromosome into a vector. The actual start location and length of each bin on chromosomes at 1 bp were omitted to reduce the length of the summarized vector. Consequently, the vector could not represent the actual scale and location of indel events. However, the adjusted log_2_ ratio in the CNR file from the CNVkit^9^ program was already adjusted to represent the length of the bin, and the indel events targeted in this study are of very large scale, occupying whole chromosome arms. Therefore, we determined that some distortion in the summarized vector is acceptable in exchange for accurate identification of arm deletion.

For the same reason, the TCGA data used as a validation set were preprocessed using the relative order of the bins and average log_2_ values. Moreover, if the summarized vector was made to represent actual scale and location, it would be ten millions of components in length for a single chromosome. Input of such a long vector would require too much computing power and data to train the CNN network.

As described above, we identified one false positive in the FISH test, suggesting that methods based on NGS could yield more accurate and objective results than conventional FISH.

In summary, this study showed that targeted NGS testing can accurately detect 1p/19q co-deletion, suggesting that this method represents a promising approach for classifying glial tumor. In addition, we applied DL to identify the co-deletion. This method yielded reproducible result in a validation set collected and processed at a different institution on a different platform. This result proves the robustness of the DL, and encourages application of DL to future NGS-based genomic analyses.

## Materials and Methods

### Samples

This was a retrospective study. The study initially enrolled 20 cases of oligodendroglial tumors (10 oligodendrogliomas and 10 anaplastic oligodendrogliomas), for which 1p/19q FISH results were available, between January 2015 and December 2016 at the Asan Medical Center, Seoul, Korea (Table 1). Oligodendroglial tumors were selected to detect the 1p/19q co-deletion because this tumor is known to harbor the 1p/19q co-deletion according to a recent up-dated classification^1^. Each deletion of 1p and 19q should occur at the chromosome arm level in a sample. The targeted NGS panel did not cover the whole chromosome; therefore, chromosome 1p deletion and 19q deletion were defined as complete segmental loss covered by NGS panel within chr1:1-125000000 and chr19:26500001-59128983, respectively, based on the hg19 human reference genome. Partial segmental loss within regions was classified as negative. Clinical information including age at diagnosis, tumor location, *IDH1* Sanger sequencing results, and pathologic diagnosis was gathered from pathology reports. Negative control samples (n = 40) were selected randomly from 1500 targeted NGS data of Asan-CCGD (Asan Center for Cancer Genome Discovery), Asan Medical Center, Seoul, Korea. The study was approved by the institution review board of Asan Medical Center. The institution review board waived the requirement to obtain informed consent. All methods were performed in accordance with relevant guidelines and regulations.

**Table 1:** Pathologic and demographic features of 20 cases.

No	Diagnosis	FISH (1p36/19q13)	*IDH1* by Sanger sequencing	Location	Sex	Age (yrs)
1	oligodendroglioma	positive	R132H	frontal	M	39
2	oligodendroglioma	positive	R132H	temporal	M	56
3	oligodendroglioma	positive	R132H	frontal	F	62
4	oligodendroglioma	positive	Negative	frontal	F	42
5	oligodendroglioma	positive	R132H	frontal	M	34
6	oligodendroglioma	positive	R132H	frontal	F	55
7	oligodendroglioma	positive	R132H	frontal	M	40
8	oligodendroglioma	positive	R132H	temporal	F	63
9	oligodendroglioma	positive	NA	frontal	M	41
10	oligodendroglioma	positive	NA	frontal	F	29
11	anaplastic oligodendroglioma	positive	NA	frontal	F	45
12	anaplastic oligodendroglioma	positive	NA	frontal	M	65
13	anaplastic oligodendroglioma	positive	R132H	frontal	F	70
14	anaplastic oligodendroglioma	positive	Negative	temporal	M	63
15	anaplastic oligodendroglioma	positive	NA	frontoparietal	M	58
16	anaplastic oligodendroglioma	positive	NA	frontal	F	52
17	anaplastic oligodendroglioma	positive	NA	frontal	M	54
18	anaplastic oligodendroglioma	positive	NA	frontal	F	66
19	anaplastic oligodendroglioma	positive	NA	frontotemporal	F	51
20	anaplastic oligodendroglioma	positive	NA	frontal	F	48

FISH, *in situ* hybridization; NA, not available; M, male; F, female.

### DNA extraction

Matched slides stained with hematoxylin and eosin (H&E) from each formalin-fixed and paraffin-embedded (FFPE) tissue sample were reviewed by two pathologists (H.S.H and S.J.N). The tumor area was marked to determine tumor purity and to guide tumor DNA extraction. Genomic DNA (gDNA) was extracted according to our previous report^19^. Briefly, 2 ~ 5 sections (6 μm thick) from the indicated area in each FFPE tissue were obtained. After de-paraffinization with xylene and ethanol, gDNA was isolated using the NEXprep FFPE Tissue Kit (#NexK-9000; Geneslabs, Seongnam, Korea). Quantification was performed using the Qubit™ dsDNA HS Assay kit (Thermo Fisher Scientific, Waltham, MA, USA).

### DNA library construction and high-throughput sequencing

Targeted NGS was performed using MiSeq (Illumina, Inc., San Diego, CA, USA) with OncoPanel AMCv3 (OP-AMCv3, developed in-house by Asan-CCGD) to include the exons of 199 genes (575,147 bp) and partial introns from 8 genes often rearranged in cancer (209,397 bp) to detect fusion genes and additional small (10,534 bp) specific single nucleotide polymorphism loci for CNV analysis. Overall, the panel covered 823,971 bp. A DNA library was prepared as described in our previous report using the S1 method^20^; briefly, gDNA shearing with S1 enzyme, end repair, A-tailing, and ligation with the TruSeq adaptor using the SureSelect XT reagent kit (Agilent Technologies, Santa Clara, CA, USA). Each library was constructed with sample-specific barcodes 6 bp in size and quantified using the Qubit kit. Eight libraries were pooled (yielding a total of 720 ng) for hybrid capture using the Agilent SureSelect XT custom kit (OP-AMCv3 RNA bait, 1.2 Mb; Agilent Technologies). The concentration of the enriched target was measured using quantitative polymerase chain reaction (qPCR; Kapa Biosystems, Inc., Woburn, MA, USA). DNA libraries that passed quality checks were sequenced using MiSeq.

### Genomic data analysis

Sequenced reads were mapped to the human reference genome (NCBI build 37) using the Burrows-Wheeler Aligner (version 0.5.9)^21^ with default options. PCR-duplicates were removed using the Picard tool. Then de-duplicated reads were realigned at known indel positions using GATK IndelRealigner, and base quality was recalibrated with GATK TableRecalibration^22–24^. Somatic mutations of single nucleotide variants and short indels were called in tumor tissue with matched normal tissue using MuTect (1.1.7)^25^ and SomaticIndelocator in GATK, respectively. Germline variants from somatic variant candidates were filtered out using the common dbsnp database (build 141; found in ≥1% of samples) and a panel of normal samples. Filtered somatic variants were annotated with the Variant Effect Predictor (v79), and then converted to MAF file using vcf2maf (v1.6.12).

Quality checks for fastq files were performed using FastQC (http://www.bioinformatics.babraham.ac.uk/projects/fastqc/). Quality checks for analysis-ready BAM files, including total read number, % PF reads, % selected bases, mean target depth, % target not covered, % target bases covered 30×, and % duplication for cleaned BAM files, were performed using the calculateHSMetrics and MarkDuplicates modules of the Picard package. Integrative Genomics Viewer (v2.4) was used to view the BAM file^26^.

### Copy number analysis

Copy number ratio (log_2_) was obtained using the CNVkit^9^. BAM files generated by the pipeline described were used as input files for CNVkit using default parameters without normal tissue. The copy number ratios were obtained and used for downstream analysis. Each bin’s adjusted log_2_ ratio, chromosomal number, and relative order in that chromosome were extracted from the CNR file. By arranging the adjusted log_2_ ratio value according to the bin’s relative order, each chromosome was summarized as a vector. The vectors summarizing chromosomes 1 and 19 were 1869 and 649 dimensions in length, respectively. For each case, the CNV plots for chromosomes 1 and 19 were plotted from these vectors. All CNV plots obtained using the log_2_ ratio were reviewed manually by two pathologists (H.S.P. and C.O.S.) to determine the presence of the 1p/19q co-deletion.

### Fluorescence *in situ* hybridization

FISH was performed on FFPE blocks using probes for 1p36 and 19q13 (Vysis, Downers Grove, IL, USA). Signals were evaluated for each locus-specific FISH probe on an Olympus BX51TRF microscope (Olympus, Tokyo, Japan) equipped with a triple-pass filter (DAPI/Green/Orange; Vysis), and at least 50–100 non-overlapping nuclei containing a minimum of two control signals with intact morphology were evaluated. No target signal or a single signal was interpreted as a deletion, and losses with any disproportion of centromere and target signals in a nucleus with more than one 1p36 or 19q13 signal (ratio 3/2, 4/3, 4/2, 5/3, etc.) were scored FISH imbalances. Because FISH imbalances often, but not always, correspond to LOH, combined criteria were used, defining 1p and 19q deletion as a combined target-to-control signal ratio <0.75 or cut-off of a nucleus with a 1 or 0 target signal >50%.

### *IDH1* sequencing

The genomic region spanning codon 132 of *IDH1* was amplified by PCR with primers 5′-TGAGAAGAGGGTTGAGGAGTTC-3′ (forward) and 5′-CACATACAAGTTGGAAATTTCTGG-3′ (reverse). The amplicon was sequenced with the forward primer using the BigDye Terminator v3.1 Cycle Sequencing Kit (Applied Biosystems, Foster, CA, USA).

### External validation using The Cancer Genome Atlas (TCGA) data set

Low-grade glial tumors (n = 426) were used for independent external validation of the DL model for detection of 1p/19q co-deletion. Copy number (CN) log_2_ data generated using Affymetrix SNP Array 6.0 platform were downloaded from The Cancer Genome Atlas (TCGA). CN probes were extracted, and the CN log_2_ ratio for tumor-matched normal tissue was calculated for each sample. The processed log_2_ CN data were reformatted to correspond to the targeted NGS data, i.e., chromosome 1 log_2_ CN data were reformatted as 1896 × 1 vectors, and chromosome 19 log_2_ CN data were reformatted as 649 × 1 vectors. Bins in chromosome 1 and 19 were gathered into 1896 and 649 groups, respectively, according to their relative order on the chromosome, and reformatting was performed by averaging the corresponding groups. The representative log_2_ ratio value for each group was calculated by the averaging log_2_ value of the bins corresponding to that group. Vectors from reformatted CN data were plotted in the same manner as CNV plots made from targeted NGS data. All CNV plots were reviewed manually by two pathologists (H.S.P. and C.O.S.) to determine the presence of the 1p/19q co-deletion.
